# Reciprocal Interactions between Lactoferrin and Bacterial Endotoxins and Their Role in the Regulation of the Immune Response

**DOI:** 10.3390/toxins2010054

**Published:** 2010-01-08

**Authors:** Daniela Latorre, Patrizia Puddu, Piera Valenti, Sandra Gessani

**Affiliations:** 1 Department of Cell Biology and Neurosciences, Istituto Superiore di Sanità, Viale Regina Elena 299, Rome, Italy; 2 Department of Public Health Sciences, Sapienza, University of Rome, Italy

**Keywords:** lactoferrin, lipopolysaccharide, inflammation, immune response

## Abstract

Lactoferrin (Lf), an iron-binding glycoprotein expressed in most biological fluids, represents a major component of the mammalian innate immune system. Lf’s multiple activities rely not only on its capacity to bind iron, but also to interact with molecular and cellular components of both host and pathogens. Lf can bind and sequester lipopolysaccharide (LPS), thus preventing pro-inflammatory pathway activation, sepsis and tissue damage. However, Lf-bound LPS may retain the capacity to induce cell activation via Toll-like receptor 4-dependent and -independent mechanisms. This review discusses the complex interplay between Lf and LPS and its relevance in the regulation of the immune response.

## 1. Introduction

Lipopolysaccharide (LPS), a major constituent of the Gram-negative bacteria outer membrane, is one of the most potent inducers of the innate immune response. Recognition of various form of LPS from different strains of Gram-negative bacteria triggers a signaling cascade that results in the release of pro-inflammatory mediators, such as cytokines and chemokines, as well as small molecules, such as lipid mediators and reactive oxygen species [1]. LPS is known to be capable of initiating the morbidity and mortality associated with Gram-negative sepsis, as well as the modulation of myriad other host innate inflammatory responses. Specifically, LPS has been characterized as the 'prototypical stimuli' for host activation through myeloid cells (neutrophils, monocytes, macrophages) and non-myeloid cells (fibroblasts, platelets), as well as other innate host defense mechanisms, such as serum complement, and specific components within the intrinsic coagulation pathway.

LPS is ubiquitous within our environment, *in vivo* and *in vitro*, and can express potent bioactivity in extremely small amounts [2]. This bacterial component is a complex molecule consisting of three parts: a core oligosaccharide, a distal hydrophilic O side chain, and a highly conserved lipid A portion [3]. The lipid A moiety is the main pathogen-associated molecular pattern of LPS, and is responsible for its toxic proinflammatory properties. Using the C3H/HeJ mouse strain, which is known to have a defective response to LPS, it has been demonstrated that the Toll-like receptor 4 (TLR4) is an important sensor for LPS [4]. LPS stimulation of mammalian cells occurs through a series of interactions with several proteins including the LPS binding protein (LBP), CD14, MD-2 and TLR4 [5]. LBP is a 60kDa acute-phase serum protein which directly binds to LPS and facilitates the association between LPS and CD14. Since the discovery of the LBP/CD14 host activation pathway [6,7] it has become increasingly clear that the release of numerous inflammatory mediators and the expression of cell adhesion molecules necessary for inflammation occur in response to LBP and/or CD14 complexed with microbial components [8,9]. After initial LBP-LPS binding, the next step toward host cell activation is the transfer of LBP-LPS complex to either soluble or membrane-bound CD14. It has been shown that aggregate forms of LPS initially bind LBP, which then facilitates the transfer of separated, monomeric LPS to either membrane CD14 (mCD14) or soluble CD14 (sCD14), found in serum [8,9]. mCD14-positive cells (usually cells of myeloid origin) contain mCD14 as a glycosylphosphatidylinositol-anchored membrane protein, whereas the sCD14/LPS complex is required for CD14-negative cell (e.g., endothelial and certain epithelial cells) activation by LPS [10,11]. CD14 facilitates the transfer of LPS to the TLR4/MD-2 receptor complex and modulates LPS recognition. MD-2, a small secreted protein which comprises part of the cell surface receptor complex, is absolutely required for the TLR4-mediated cellular response to LPS [12]. After activation by LPS, TLR4 recruits adapter molecules such as MyD88, Mal, Trif, and Tram within the cytoplasm of cells to propagate a signal [5]. These adapter molecules in turn activate other molecules within the cell, including protein kinases IRAK1, IRAK4, TBK1, and IKKi, to amplify the signal, and result in the induction or suppression of genes that orchestrate the inflammatory response. LPS/TLR4 signaling can be separated into MyD88-dependent and MyD88-independent pathways, which mediate the activation of proinflammatory cytokines and type 1 interferon (IFN), respectively [5].

The outcome of Gram-negative infections is dependent not only by an individual’s ability to recognize endotoxin and respond to its presence but also by numerous phenomena that inactivate endotoxin and/or prevent harmful reactions to it [13]. Until now, many detoxification mechanisms have been described acting in different body compartments, including proteins that facilitate LPS sequestration or prevent endotoxin interaction with its receptors. In this respect, lactoferrin (Lf), an 80 kDa non-heme-iron binding glycoprotein, represents one of the most efficacious mechanisms of LPS neutralization, both in tissues and secretions, activated by the innate response in peripheral tissues during the inflammatory processes. Lf is physiologically found in exocrine secretions of mammals, in particular in milk and fluids of the digestive tract, and is abundantly released by exocrine glands of mucosa and by neutrophils during inflammation [14]. Lf is a key element in the host defense system. This assumption is mainly based on the antimicrobial properties of this molecule, which include iron sequestration (bacteriostatic activity), direct lytic activities (bactericidal activity), and/or impaired binding of microbes to host cells [15]. More recently, however, it has becoming evident that its protective effect also extends to the modulation of the host response to infections. Depending on the immune status of an individual, Lf can have anti-inflammatory properties which are mostly explained by its capacity to interact with exogenous proinflammatory molecules, mainly LPS [16] and its CD14 receptor [17], as well as CpG bacterial DNA [18], thus decreasing the immune response and preventing septic shock and damage to tissues. However, Lf can also favor the activation, differentiation, and proliferation of immune cells, and this promoting activity has been related to a direct effect of Lf on immune cells [19,20].

## 2. Structure of Lf and Molecular Basis of Lf-LPS Interaction

Lf is a monomeric, highly cationic (pI 8.4-9.0) glycoprotein with a single polypeptide chain of about 690 amino acid residues. Crystallographic analysis of Lf from different species has revealed a highly conserved three-dimensional structure, but with differences in detail between species [21]. The polypeptide is folded in two symmetric globular structures named the N and C lobes, which are linked by a short α-helix. Non-covalent interactions, mostly hydrophobic, provide a cushion between the two lobes, with C-terminal helix playing a large part. Both lobes have the same fold, consistent with their sequence homology of ~ 40%. Each lobe, two *α/β* domains, referred to as N1 and N2 or C1 and C2, encloses a deep cleft within which is the iron binding site. Each site binds at a remarkably high affinity (K_d_ 10^22^ M), but reversibly, one Fe^3+^ ion [22].

**Figure 1:**
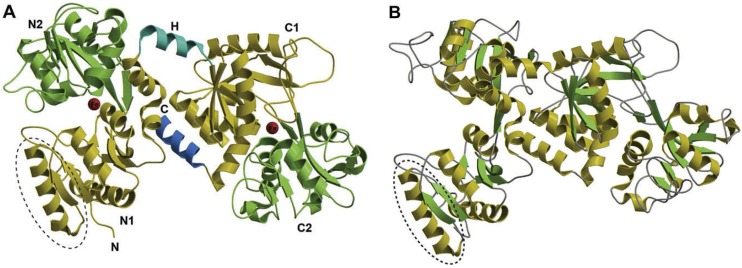
Polypeptide folding of human lactoferrin. Structure of the iron-bound (holo) form (A) and iron-free (apo) form (B) of Lf reprinted with permission from [22].

The two lobes, four-domain structure, provides the key to understanding the dynamic properties of Lf (Figure 1). Indeed, two structures have been observed for this molecule: a closed conformation (Figure 1A), mainly observed with the iron-saturated molecule (holo-Lf), and an open conformation, originally described for the iron-free Lf (apo-Lf) (Figure 1B). The conformational transition could be involved in basic functions such as transportation and catalysis. According to crystallographic data, the domains move essentially as rigid bodies that close over the bound metal or open to release it. Metal binding and release are facilitated by the flexibility of the apo form, and strong retention by the relative rigidity of the holo form. Recently, Lf has been described as a molecule with a double face, composed by an internal portion, highly conserved between species and endowed with iron binding capacity, and an external surface strongly cationic and prone to interact with a number of negatively charged macromolecules. The positive charge, mainly concentrated at the *N*-terminus (amino acids 1-7), in the first helix (amino acids 13-30) and in the region that connects the two lobes, is thought to be crucial for the majority of Lf activities, including immunomodulation and LPS binding [22]. In particular, the first helix includes the major portion of the lactoferricin domain, a potent bactericidal peptide [23], and the *N*-lobe that participates as binding domain in Lf-pathogen interactions [24] as well as ceruloplasmin binding [25].

Structurally, Lf contains a highly basic arginin-rich region close to the *N*-terminus which binds to a variety of anionic biological molecules [26,27]. Human Lf (huLf) binds specifically and with a high affinity (K_d_ = 2-5 nM) to the lipid A moiety of bacterial LPS [16,28], sCD14 and the sCD14/LPS complex [17], heparin, or cell-surface heparan sulphates [26,29,30]. HuLf binding to anionic molecules mostly relies on two *N*-terminal basic clusters (residues 1 to 5 and 28 to 34). Specifically, two binding sites for *Escherichia Coli* 055B5 LPS have been found on human Lf: a high affinity (K_d_ 3.6 ± 1 nM) site located at the *N*-terminus, and a low-affinity (Kd 390 ± 20 nM) site at the *C*-terminus, which is exposed at high protein concentrations [28]. Several studies indicate that Lf is capable of destabilizing the outer membrane of Gram-negative bacteria upon its binding to LPS exposed on the bacterial surface, promoting LPS release and bacterial killing through osmotic damage [31]. It has been reported that LPS release *in vitro* may occur without a direct interaction of Lf with bacteria but requires sequestering of Ca^2+^ by Lf. Calcium binding to Lf destabilizes bacterial membrane while has a marked stabilizing effect on the protein structure towards thermal and chemical denaturation [32]. All Lfs are glycosylated with some differences between species, having huLf three and bovine Lf (bLf) five potential *N*-glycosylation sites [22]. Studies characterizing the Lf structure have demonstrated that only a part of these potential sites are usually glycosylated, two in huLf and four in bLf [33,34]. Although glycosylation has no influence on Lf folding, most of the glycosylation sites are exposed on the external surface of the molecule and have been supposed to play a role in Lf interaction with viruses [15], toxins [35], sialic acid-binding immunoglobulin superfamily lectins [36] and C-type lectin receptors on immune cells [37,38].

The interaction of bacterial endotoxins with Lf has been investigated at a biophysical level. The formation of LPS-Lf complexes occurs through electrostatic interactions. It was shown that Lf binds to the phosphate group within the lipid A moiety and induces a rigidification of the acyl chain of LPS. The secondary structure of Lf was, however, not changed [39]. Binding saturation was found to lie at a [Lf]:[Lipid A] ratio of 1:3 to 1:5 M and promotes the conversion of the molecular shape of lipid A from a conical form (active) into a cylindrical form (inactive), in keeping with previous studies suggesting that the conical shape of lipid A is a prerequisite for its endotoxic activity [40,41]. Lf was shown to intercalate into phospholipid liposomes and to block the LBP-mediated intercalation of LPS, suggesting that conversion from an active to inactive form occurs at the plasma membrane level [39].

In addition, Lf may act by interfering with the access of endotoxin to its cell surface receptor. Indeed, evidence has been provided that huLf inhibits the interaction of LPS with CD14 on monocytes/macrophages by competition with the LBP, which binds to the lipid A portion of LPS, thus mediating the transfer of LPS to CD14 [28]. In particular, Elass-Rochard and colleagues [28], by using a mutated huLf demonstrated that the loop region containing amino acids 28 to 34, located in the *N*-terminal domain I, is essential for the high-affinity interaction with LPS. This region is also present in lactoferricin, which *in vitro* suppresses the release of IL-6 from monocytic THP-1 cells stimulated with LPS [42].

Subsequent studies led to the identification of a cationic region, the GRRRR *N*-terminal sequence (amino acids 1-5), which plays a crucial role in the competition of human LBP for LPS binding [43]. Specifically, it has been demonstrated that recombinant huLf lacking residues 1 to 5 does not inhibit the LBP-mediated binding of LPS, suggesting that these Lf residues may interact synergistically with residues 28 to 34 as a cationic cradle to bind LPS.

Overall, LPS-binding property of Lf plays an important role in the immunomodulatory activity of this molecule offering a dual advantage. In fact, from one side, Lf can sequester LPS, thus inhibiting the excessive host’s response to endotoxin challenge, and from the other, can take advantage of the LPS bound to its molecule to trigger an immune response engaging specific LPS receptors.

## 3. Lf Interference with LPS Inflammatory Activity

The endotoxin-chelating properties of Lf and its ability to compete with LPS receptors account, in part, for the antinflammatory activity of this protein. Several *in vitro* studies have demonstrated that Lf can inhibit, in a concentration- and time-dependent manner, a number of LPS-induced effects (Table 1). Although the mechanisms responsible for this inhibitory activity have not been fully elucidated, at least some may be ascribable to Lf capacity to avidly bind LPS, thus blocking its interaction with cellular membranes or compete with LPS for binding to a common receptor.

In this respect, Baveye and co-workers demonstrated that huLf, at LPS serum concentrations observed in pathological conditions, blocks the LPS-induced production of oxygen free radicals by competing with L-selectin, a serum-independent LPS receptor in neutrophils, for LPS binding [44]. Similarly, Elass-Rochard and colleagues [43] showed that huLf also prevents the LBP-mediated binding of LPS to the CD14 receptor. However, maximal inhibition of LPS interaction with the cell occurs when huLf and LBP are simultaneously added or pre-incubated together prior to their addition to the cultures, but not when huLf is added after LBP interaction with LPS has occurred. These results suggest that huLf competes with LBP for LPS binding, and this competition negatively affects the subsequent interaction of LPS with CD14 [43]. The alignment of the *N*-terminal sequences of huLf and LBP shows similarity between amino acid residues of these proteins. Notably, a structural motif of three basic amino acids separated by one hydrophobic amino acid is present in both huLf and LBP. Despite these shared features, LBP and huLf exhibit antagonistic effects by favouring or inhibiting LPS interaction with CD14, respectively. In keeping with these results and with the antinflammatory activity of Lf, it has been shown that the LPS-triggered release of IL-1, IL-6, IL-8, TNF-α in a variety of human and mouse cell types is inhibited in the presence of Lf [45,46,47]. In particular, Haversen and co-workers [46] showed that in human monocytic cell line Lf was internalized into cells and detected in the nucleoli. In keeping with this finding, Lf was showed to decrease the LPS-induced binding of NF-κB to TNF-α promoter. Lactoferricin-derived peptides were also reported to down-modulate TNF-α production in human PBMC by changing the aggregate structure of LPS or lipid A into a multilamellar form [48]. Furthermore, it has been reported that huLf down-modulates the LPS-induced expression of some adhesion molecules, e.g., ICAM-1 and E-selectin, in endothelial cells [17]. This effect was shown to rely on the huLf capacity to bind specifically and with high affinity to sCD14 and to LPS-CD14 complexes. This observation suggested that Lf can modulate the recruitment of immune cells to inflammatory sites by down-regulating the adhesion of leukocytes to endothelial cells. In keeping with this hypothesis, it has been reported that Lf inhibits LPS-induced expression of IL-8, and competes with this chemokine for its binding to proteoglycans of endothelial cells [49]. Lf has also been reported to protect intestinal cells against LPS-induced mucosal damage [50] as well as to inhibit LPS-induced proliferation, prostaglandin E_2_ production, and cyclooxygenase (COX)-2 and matrix metalloproteinase (MMP)-9 expression in PBMC [51] via unknown mechanisms. Lastly, Lf released from LPS-activated neutrophils and apo-Lf have been reported to exert protective effects on neutrophil priming for enhanced superoxide production [52,53].

**Table 1:** Lactoferrin protective activity on *in vitro* LPS-induced effects.

Cell	Lf type	LPS-induced functions	Suggested mechanism	References
Mouse RAW 264.7	huLf, bLf	Cytokine production (TNF-α IL-1β, IL-6, IL-8)	Inhibition of NF-kB activation	[42,45-47,49]
Human THP-1				
Human Mono Mac 6				
HUVEC	huLf	Cytokine production (IL-8)	Interaction with sCD14/LPS complex	[49]
Human PBMC	Lfcin-derived peptides	Cytokine production (TNF-α)	LPS inactivation via structural changes	[48]
Human THP-1	huLf, bLf, Lfcin B	Cytokine production (IL-6, IL-1, TNF-α)	Not determined	[42,47]
Primary human monocytes				
Human PBMC				
HUVEC	huLf	Endothelial adhesion molecule expression	Interaction with sCD14/LPS complex	[17]
Human neutrophils	huLf	Hydrogen peroxide production	Inhibition of LPS binding to L-selectin	[44]
Human neutrophils	Neutrophil released Lf	Priming for enhanced superoxide production	LPS sequestration	[52,53]
	ApoLf*			
Bovine PBMC	bLf	Proliferation	Not determined	[51]
		PGE_2_ production		
		COX-2 and MMP-9 expression		
Human CaCo2	huLf	Intestinal mucosa damage	Not determined	[50]

* Lf source not indicated in the original article.

This Table summarises the *in vitro* results describing the Lf inhibitory actions on LPS-stimulated cell responses. HUVEC, human umbilical vein endothelial cells; PBMC, peripheral blood mononuclear cells; huLf, human lactoferrin; Lfcin, lactoferricin; bLf, bovine lactoferrin; PGE_2_, prostaglandin E_2_; COX-2, cyclooxygenase (COX)-2; MMP-9, matrix metalloproteinase (MMP)-9.

The capacity of Lf to modulate the LPS-induced inflammatory process has been also well documented *in vivo*. Indeed, several studies [54,55,56,57,58,59] have demonstrated that Lf administration protects animals against sub-lethal doses of LPS (Table 2). Interestingly, the optimal protection against induced septicaemia required a 12 to 24 h pre-injection of Lf, suggesting that this protein may act by mechanisms in addition to simple LPS scavenging [54]. Furthermore, mice previously treated with Lf did not develop hepatitis [60], arthritis [61], diarrhea [58] and preterm delivery [62,63,64] after LPS challenge. The exact mechanisms by which Lf exerts preventive and/or therapeutic potential are not yet known, however, some of the effects could be due to Lf capacity to neutralize LPS *in vivo*. In keeping with the antinflammatory effect of Lf observed in *in vitro* studies, serum levels of LPS-induced pro-inflammatory factors such as IL-6, TNF-α and nitric oxide were found significantly reduced in Lf-treated animals in comparison with untreated controls after LPS inoculation [61,65,66,67]. In addition, growing evidence indicates that progression of systemic inflammatory response syndrome into sepsis is due to the cellular damage and death induced by acute inflammatory response. In this respect, Kruzel and colleagues have recently reported that Lf protects against oxidative stress-induced mitochondrial dysfunction and DNA damage, both in cell culture and within an animal model of endotoxemia [68].

**Table 2:** Protective effects of lactoferrin on LPS-triggered pathologies *in vivo.*

Animal	Lf source	Administration	LPS-triggered effects	Lf activity	References
Mice, Piglets	bLf, huLf, Lfcin-derived peptides	i.v., i.p., p.o.	endotoxin lethal shock	survival	[54,55-57]
Mice	bLf, huLf	i.p.	preterm delivery	prevention	[62-64]
Mice	huLf	i.v.	hepatitis	protection	[60]
Rats, Mice	bLf, huLf	i.v., p.o., i.p.	TNF-α, IL-6, IL-10, NO production	decreased	[61,65-67]
Rats	bLf	p.o.	arthritis and hyperalgesia	prevention	[61]
Mice	bLf	i.p.	diarrhea	prevention	[58]
Mice	huLf	i.p.	liver mitochondrial dysfunction	protection	[68]
Rats	bLf, huLf	i.p.	albumin extravasation, neutrophilia	prevention	[59]

This table reports experimental evidence of Lf beneficial effects in animal models challenged with LPS. huLf, human lactoferrin; bLf, bovine lactoferrin; NO, nitric oxide; i.v., intravenous; i.p., intraperitoneal; p.o., per os.

## 4. Biological Activity of Lf-Bound LPS: TLR4 -Dependent and -Independent Effects

Despite the well recognized activity of Lf as a powerful scavenger of endotoxins, some studies documented that Lf-bound LPS retains the capacity to stimulate mouse and human cells. In particular, it was reported that Lf-LPS complexes can still prime human monocytes and stimulate B lymphocyte proliferation [53].

Furthermore, Na and co-workers reported that when LPS and purified Lf were mixed, and formed a complex, induction of proinflammatory mediators or tolerance, rather than inhibition of LPS challenge, were observed in RAW 264.7 cells and peritoneal macrophages harvested from C3H/HeN mice [69]. Similarly, Chodaczek and colleagues have showed that Lf complexed to monophosphoryl lipid A can exert adjuvant activity in mice immunized with a suboptimal dose of sheep red blood cells or ovalbumin, thus increasing their humoral immune response [70]. Comparative studies carried out with LPS responsive and LPS hypo-responsive mice demonstrated a strong dependency of the Lf-LPS complex triggered signals on TLR4, leading to the conclusion that the immunomodulatory properties of Lf could be due, at least in part, to LPS binding [69]. Lf binds to the lipid A portion of LPS via charge-charge interaction. The portion of Lf that binds to anionic molecules, including lipid A, is limited to its *N*-terminus arginine rich domain [27]. Thus, it is likely that bound LPS can still expose the unbound part of lipid A that is recognized by LPS receptors such as TLR4. Such a Lf-LPS complex recognition would result in macrophage activation [69]. In this regard, it has been reported that Lf-bound LPS retains clotting capacity in a conventional Limulus assay (LAL), the standard method for detection of endotoxin contamination [39]. Of note, the lipid A backbone is also the epitope being recognized in this assay, thus explaining why the Lf-LPS complex is found to be LAL positive [39,69]. Collectively, these results suggest that lipid A can be recognized even after Lf-LPS complex has been formed, and that this complex retains the capacity to activate macrophages through TLR4. Lf preparations experimentally used may contain some LPS. However, due to the extremely high capacity of Lf to form complexes with LPS through its lipid A moiety, it is conceivable that the little amount of LPS commonly detected in Lf preparations, is all bound to Lf molecule. In keeping with this assumption, we have previously demonstrated that low concentrations of LPS corresponding to the amount naturally present in Lf batches is per se not capable to induce type 1 IFN secretion in murine peritoneal macrophages, in contrast with the capacity of Lf to induce it. However, it has been reported that LPS binding to Lf may contribute to Lf biological activity by favouring its interaction with cell surface receptors [71]. Thus, LPS may represent an important structural component of Lf molecule, likely involved in its stabilization or favouring its interaction with receptors and accessory molecules. 

Despite these observations, the intimate relationship between Lf and LPS does not completely account for the different biological activities ascribed to this molecule. In keeping with these results, we have reported that the capacity of Lf to induce a type 1 IFN mediated antiviral state, but not TNF-α production, relies on the function of TLR4 in responding cells [72]. Our results showing that TLR4 is not essential for Lf-induced production of TNF-α by murine peritoneal macrophages, strongly suggest that this molecule induces macrophage activation via TLR4-dependent and -independent mechanisms. Accordingly, it has been recently reported that Lf-induced IL-6 secretion and CD40 expression in murine peritoneal macrophages was achieved via TLR4-independent and -dependent mechanisms, respectively, thus indicating potentially separate pathways for Lf-mediated macrophage events in innate immunity [73]. Likewise, a dichotomous nature of Lf binding to monocyte/macrophage-differentiated HL-60 cells, one being mediated by specific Lf receptors whereas the other occurring mainly via LPS receptors after formation of Lf-LPS complexes, was also reported [71].

Thus, Lf binding to LPS may represent an important aspect, but does not entirely account for all immunomodulatory effects of this molecule. This aspect could be mostly relevant in those cell types, such as the macrophages, in which TLR4 function is of critical importance in the regulation of their activity.

## 5. Concluding Remarks

**Figure 2:**
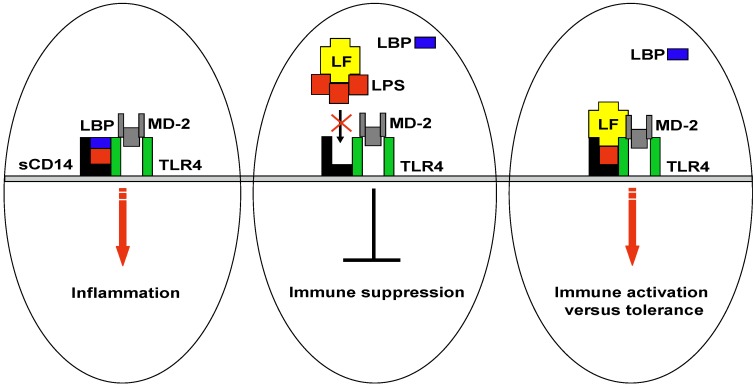
Lactoferrin interplay on LPS-induced inflammatory response. A schematic representation of Lf interaction with LPS highlighting the multitasking strategy of Lf to maintain immune homeostasis. Lf behaves as a versatile molecule by efficiently suppressing endotoxin-induced excessive immune reaction in sepsis or promoting, in particular conditions, a protective response against pathogen challenge.

Lf is a natural defence component of the innate immunity only found in mammals. This exclusive characteristic has suggested that this molecule could be involved in newborn nutrition and protection. However, in adult life, Lf continues to exert a plethora of biological activities. Its protective effects range from direct antimicrobial activities against a variety of pathogens, including bacteria, viruses, fungi and parasites, to anti-inflammatory and anti-tumour activities. These multiple functions rely not only on the capacity of Lf to sequester iron, but also on its property to interact with molecular and cellular components of both host and pathogens, including endotoxins and their receptors. In this respect, the ability of Lf to bind LPS or limit its *in vitro* interaction with LBP and sCD14 suggests that Lf behaves as a versatile molecule by efficiently suppressing endotoxin-induced excessive immune reaction in sepsis or promoting, in particular conditions, a protective response against pathogen challenge (Figure 2).

In this scenario, we can speculate that in systemic infections, when LPS is present at high concentration, Lf drastically increases in serum where successfully targets and neutralizes either LPS or sCD14 alone, as well as already assembled LPS-CD14 complexes. Moreover, in peripheral tissue infections, Lf released by recruited neutrophils sequesters LPS impeding its association with LBP and consecutively reducing the rate and sensitivity of the response. The Lf mediated LPS sequestration would result in inhibition of activated cells, including neutrophils and monocytes, followed by tissue repair.

Conversely, under physiologic conditions, Lf could form complexes with LPS originating from commensal flora. Such Lf-LPS complexes may retain the capacity to bind to specific receptors associated to TLR4 pathway either promoting tolerance, or locally stimulating low levels of cytokines/factors. These Lf effects would contribute to maintain homeostasis and keep immune cells alerted against pathogen attack.

Understanding the molecular mechanisms underlying the capacity of Lf to reduce excessive inflammation and stimulate host immune responses, as well as identifying cell targets and receptors involved in Lf immunomodulatory activities holds the promise of a better exploitation of Lf therapeutic potential.
